# Provider Bias in Family Planning Services: A Review of Its Meaning and Manifestations

**DOI:** 10.9745/GHSP-D-19-00130

**Published:** 2019-09-23

**Authors:** Julie Solo, Mario Festin

**Affiliations:** a Independent Consultant, Durham, NC, USA.; b Department of Reproductive Health and Research, World Health Organization, Geneva, Switzerland.

## Abstract

Family planning programs are guided by the principle of informed choice as well as the goal of providing a broad choice of contraceptive methods to clients. Provider bias is an important barrier to realizing this goal, but it must be clearly defined and understood to be effectively addressed. This review presents an overview of the concept of provider bias in family planning, focusing on the following issues: (1) what it is, (2) how widespread it is, (3) its underlying causes, (4) its impacts, and (5) how it can be effectively addressed. The definitions of provider bias include common themes about providers creating barriers to choice, typically based on the characteristics of either a client or a contraceptive method. However, an agreed-upon definition is lacking. Measurement of provider bias has often relied on self-reports by providers but has also included observation and use of mystery clients for supplemental data. The general trend in the data is clear: large numbers of providers impose barriers and restrictions beyond those that are in guidelines or are necessary for any medical reasons. This trend indicates the presence of bias. Providers have shown bias based on age, parity, marital status, and other criteria, with a bias against provision of various contraceptive methods to youth being the most common. Provider bias often stems from broader social norms, particularly judgments about sexual activity among youth and concerns about the impact of hormonal methods on future fertility. Little documentation of the impact of provider bias exists, although method mix skew has been identified as a possible red flag for bias. Newer approaches to address bias that have moved beyond traditional training and guidelines development to more fundamental behavior change efforts show promise, and learning from their lessons will be important. A major question is how to scale up such approaches.

## Abstract (precis)

Provider bias, including bias regarding client age, parity, and marital status, persists as an important barrier to contraceptive choice and access. Newer approaches to mitigate bias that have moved beyond training and guideline development to more fundamental behavior change show promise.

## INTRODUCTION

Family planning programs are guided by the principle of informed choice as well as the goal of providing a broad choice of contraceptive methods to clients. However, a number of barriers limit an individual's access and actual choice, including both supply and demand factors. This situation leads to high numbers of women with an unmet need for modern contraception, which has been estimated to be 214 million women in developing regions.^1^ Providing choice equally to everyone is a fundamental right (Box)^2^ and is necessary to meet the diverse needs of clients.

BOX **World Health Organization Guidance on Contraception and Human Rights**

**Ensuring human rights in the provision of contraceptive information and services**

*1. Non-discrimination in provision of contraceptive information and services*

1.1 Recommend that access to comprehensive contraceptive information and services be provided equally to everyone voluntarily, free of discrimination, coercion or violence (based on individual choice).

1.2 Recommend that laws and policies support programmes to ensure that comprehensive contraceptive information and services are provided to all segments of the population. Special attention should be given to disadvantaged and marginalized populations in their access to these services.

Source: WHO (2014).^2^

Provider bias has been reported as an important barrier to the right to choice and as a violation of the principle of nondiscrimination, particularly for people with the highest unmet need, such as adolescents and the poor. Provider bias in contraceptive services must be clearly defined and understood to be effectively addressed.

## METHODS

This review presents an overview of the concept of provider bias in family planning, including trends over time in its description and measurement as well as the ways to address it. We focused on resources that pertained to key issues in provider bias in family planning: (1) what it is, (2) how widespread it is, (3) its underlying causes, (4) its impact, and (5) how it can be effectively addressed.

We conducted searches on “provider bias” and “family planning” in the PubMed (32 resources) and POPLINE (77 resources) databases as well as Google Scholar (732 results, although many were repeats or not relevant beyond simply mentioning the term provider bias). We identified several additional sources by speaking with key informants knowledgeable on the subject, and we also looked at relevant documents on rights and medical eligibility criteria. After removing duplicate or nonrelevant sources found in the searches, we focused on the resources included in the references.

## RESULTS

### What Is Provider Bias?

A landmark paper in 1992 on medical barriers to access family planning gave visibility to the concept of provider bias, and situation analysis studies in many countries in the 1990s made the concept more concrete through measurement.^3^^,^^4^ Although frequently cited as an important barrier to choice over the years, provider bias has often lacked a clear definition. According to the *New Oxford American Dictionary*,^5^ bias is:

*prejudice in favor of or against one thing, person, or group compared with another, usually in a way considered to be unfair.*

This definition highlights the idea of bias as an attitude and also captures the concept of fairness and a human rights perspective.

Shelton et al. included provider bias as 1 of 6 types of medical barriers: (1) contraindications, (2) eligibility, (3) process hurdles, (4) who provides contraception, (5) provider bias, and (6) regulation. They explained^3^:

*These obstacles to [family planning] are considered practices which may have a medical rationale in some manner but are scientifically unjustified … Provider bias has powerful effects on the methods that clients use. A mistaken medical rationale often underlies provider bias. Such bias influences how providers present and recommend different methods.*

Although overlap and interaction exist among these barriers, in this review, we aim to separate out and explore provider bias because addressing it requires specific types of interventions.

One of the earlier definitions of provider bias in the literature came from Bertrand et al. in 1995^6^:

*This barrier includes the practice of favoring some methods and discouraging others in the absence of a sound medical rationale, as well as failing to ascertain and to respect the client's preference.*

Campbell et al.^7^ in 2006 described bias as follows:

*Service providers sometimes deny access to a family planning method as a result of their own prejudices about the method or its delivery system.*

Sieverding et al.^8^ discussed an evolution in thinking about provider bias. They explained that it was initially understood as discouraging use of certain methods by certain populations mostly due to erroneous medical rationales. Over time, a more multidimensional understanding evolved, encompassing the idea that bias could also stem from inadequate technical skills or personal beliefs. Bias can lead to limiting choice directly by not offering a particular method to a particular client, while indirectly, it can lead to a provider making assumptions and failing to fully assess a client's needs and preferences.

Bias can lead to limiting choice directly, while indirectly, it can lead to a provider failing to fully assess a client's needs and preferences.

Definitions are particularly blurry at the lines between attitudes and behaviors, which are linked but clearly different. Definitions generally tend to describe the latter, that is, the practices and actions that arise due to bias, such as restricting access to specific types of clients. A 2017 review of provider bias regarding youth noted that “provider bias can exist as both attitude and behavior.”^9^ Even if provider bias is taken to encompass both attitude and behavior, it is important to clearly delineate the underlying attitudes, whether based on cultural or religious beliefs or lack of accurate knowledge, and the actions that result from these biases and directly restrict access and choice. In reality, all people have prejudices and biases. What is important is identifying biases and trying to ensure that they do not lead to actions that restrict choice.

Discussion about the difference between implicit and explicit bias has been limited in definitions, although recent references place more emphasis on this distinction. It is important to acknowledge and understand that while some bias is conscious and intentional, some is unconscious and unintentional; both must be recognized and addressed. In looking at bias toward youth, Starling et al.^9^ explained how both explicit biased attitudes (such as belief that youth are less able to make their own decisions) and implicit subconscious beliefs (influenced by social and biographical factors) can result in biased behavior that limits access, including hostile treatment of youth, incomplete counseling, or judgmental expressions.

The definitions over time include common themes about providers creating barriers to choice, either based on the characteristics of a client or a contraceptive method. However, the family planning field lacks an agreed-upon definition.

### How Widespread Is Provider Bias?

To understand provider bias, measurement is needed. A 2006 review of barriers to fertility regulation noted “problems of quantifying barriers limit understanding of their importance.”^7^ Most often, provider bias has been measured and documented through in-depth interviews with providers self-reporting on imposing barriers. In some studies, bias is described through client-provider interactions. A number of studies have used simulated or mystery clients to explore specific types of bias, such as toward unmarried or young clients, and to supplement self-reported data on provider behavior.^10^^,^^11^ A substantial share of the data collected around provider bias pertains to what providers say they do, or in some cases, what they actually do. But fewer studies have been aimed at more clearly understanding the providers' beliefs and attitudes that lead them to impose restrictions regardless of whether such restrictions are warranted by normative guidance from scientific and programmatic experts, for example, as found in the World Health Organization's (WHO's) Medical Eligibility Criteria for Contraceptive Use.

A number of studies have used multiple methods to obtain a richer picture of provider bias. For example, a study on provider bias toward young people in Nigeria used data collected through mystery client visits and in-depth interviews. The mystery client methodology is useful for observing actual provider behavior without the risk of social desirability bias than can occur in interviews, and in-depth interviews can assess more fully why providers do what they do. This study also employed vignette-based interviews to see how providers would behave in specific situations to better understand provider decision making.^8^ A 2017 literature review for the Beyond Bias project mentioned the effectiveness of using such hypothetical clinical vignettes to measure bias and suggested their use within the project.^9^

There are different sources of data around provider bias and different ways of presenting the information. But the general trend is clear: large numbers of providers impose barriers and restrictions beyond those conveyed in normative guidelines or needed for any medical reasons. This trend indicates the presence of bias. Although we divide provider bias into 2 broad categories—client related and method related—the categories are often connected. For example, providers are typically more likely to impose age or parity restrictions on provision and use of provider-dependent methods such as long-acting reversible contraceptives (LARCs; i.e., IUDs and hormonal implants) and permanent methods (vasectomy and tubal ligation) as compared with short-acting resupply methods such as condoms or pills. However, the underlying cause of the bias differs and is guided by attitudes and judgments about methods or particular types of clients, so it is useful to separate them.

Large numbers of providers impose barriers and restrictions beyond those conveyed in normative guidelines or needed for any medical reasons.

### Client-Related Bias

**FIGURE 1:**
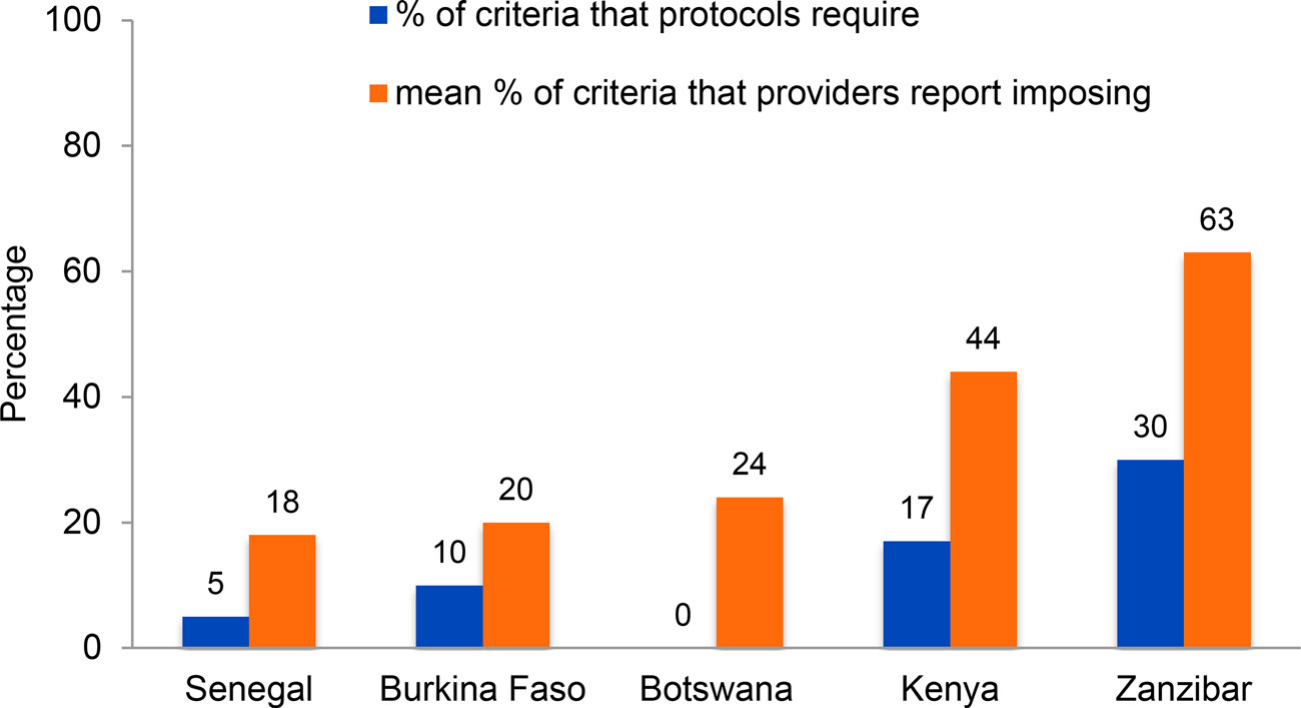
Providers Report Imposing More Eligibility Criteria Than Those Required by Protocols, Across 5 Countries

Descriptions of bias have often focused on providers imposing unjustified restrictions on use of specific methods based on age, parity, marital status, and spousal consent. In some cases, providers are following guidelines, but often many providers go beyond what is required. From 1992 to 1999, situation analysis studies gathered data to measure the extent to which providers impose various restrictions on the availability of contraceptive methods. A review of 5 studies (Botswana, Burkina Faso, Kenya, Senegal, and Zanzibar) looked at staff-imposed restrictions around marital status, spousal consent, parity, and minimum and maximum age with respect to 6 methods (oral contraceptives, condoms, IUDs, injectables, Norplant implants, and female sterilization). Providers were asked about each eligibility criterion in combination with each method. In all 5 countries, marital restrictions were imposed most commonly in prescribing IUDs and female sterilization and least commonly for condoms. A considerable proportion of providers imposed parity requirements for the provision of IUDs and injectables—not surprising at that time, given that restrictions on IUDs were actually required by policy in Burkina Faso, Kenya, and Zanzibar. To compare across countries, the review authors calculated the percentage of eligibility criteria a provider applied and then the mean score among all providers in each country. As Figure 1 shows, in each of the 5 countries, providers on average imposed twice as many eligibility criteria as were required or encouraged by national guidelines. The authors concluded^4^:

*Revised service protocols and training programs may remove the concerns about clients' well-being but addressing broader societal and cultural concerns will require more careful attention.*

#### Provider Attitudes Against Provision

A study in Malawi in 1994–1995 found that providers' attitudes had a significant impact on their practices of contraceptive provision.^12^ All providers felt that their attitudes had some effect on clients, with 61% describing this effect as large. They acknowledged that their attitudes could influence the number of new and continuing clients, who actually becomes a client, and what method the client chooses. Providers had particular issues with policies around provision of contraception to adolescents, with more than two-fifths saying they would not be comfortable providing services to young, unmarried women without children. These attitudes mostly arose from 2 beliefs: that providing these services would encourage promiscuity and contribute to the spread of HIV. Many providers did not know about or did not agree with new policies that would permit a woman to get tubal ligation whenever she wanted it, irrespective of parity, with one-third believing that tubal ligations should only be performed on women with at least 4 children.

Providers had particular issues with policies around provision of contraception to adolescents.

#### Provider Bias Against Age

Analysis of data from the 1996 Tanzania Service Availability Survey found that high proportions of providers restricted eligibility by age, with between 79% and 81% of medical aides, trained midwives, maternal and child health aides, and auxiliary staff imposing age restrictions for the pill. Among all providers, 10%–13% reported that there was at least 1 modern method they would never recommend.^13^ Data from Lesotho also showed that restrictions were common based on parity, marital status, and age, with more than 60% of providers imposing parity restrictions for IUDs and injectables.^14^

More recently, data from the Urban Reproductive Health Initiative (URHI), an initiative implemented in Kenya, Nigeria, Senegal, and Uttar Pradesh, India, between 2010 and 2015, explored the issue of provider bias.^15^^–^^18^ This analysis used facility-level data collected in all 4 countries by the Measurement, Learning & Evaluation Project as part of an evaluation of URHI. Results were reported somewhat differently in each paper, so the analysis is more useful for discerning overall trends rather than making direct comparisons among countries (see Table for detailed results). The theme of minimum age being the most prevalent restriction generally holds true for most countries and most types of providers. However, minimum age bias was assessed somewhat differently in each country. In Kenya, minimum or maximum age bias was noted if providers reported refusing methods to women within the range allowed by guidelines for all methods (15–49 years). In Nigeria, minimum age bias was defined as providers indicating the minimum age they would offer a method to a client as 15 years or older. In Uttar Pradesh, a minimum age of 18 was considered as a barrier—this more conservative definition was based on the legal age at marriage in India. In Senegal, providers were asked to report the minimum age a client had to be for them to offer a method; if they did not report a minimum age, they were considered as not restricting by age. Regardless of how it was assessed, however, the majority of providers across countries demonstrated a minimum age bias. For example, in Kenya 58% of providers would impose minimum age requirements for 1 or more methods, and in Nigeria minimum age restrictions were imposed by between 70% and 93% of providers across method and provider type. Restrictions based on parity, marital status, or age were more likely to be imposed for longer-acting methods such as the IUD as compared with pills or condoms. Trends were less consistent in terms of public/private differences and differences between cadres.

**TABLE.** Selected Data From the Urban Reproductive Health Initiative Surveys: Providers' Self-Reported Restrictions

Country (Reference)	Sample size	Age	Parity	Marital status
Uttar Pradesh, India (Calhoun et al.^18^)	1,751 (406 public, 1,345 private)	30% of doctors restricted access to pills based on a minimum age; more than 70% restricted access to sterilization and IUD based on a minimum age requirement. More than 70% of nurses and midwives restricted the IUD based on a minimum age. Approximately 50% of doctors said they restrict access to injectables based on a minimum age.	90% of providers restricted access to female sterilization and IUD based on the client's parity. 65% of these doctors required the client to have 1 child, and 63% of TBAs required 2 children for an IUD. Government of India guidelines require that a client have at least 1 child, but 83% of doctors required a client to have at least 2 children for female sterilization. Parity restrictions were imposed for pills by 66% of nurses versus only 20% of doctors and 25% of TBAs. Almost 50% of these providers required that a client have 2 children.	Nearly 99% of doctors restricted access to sterilization based on marital status, which may be related to Government of India guidelines requiring women to be ever-married. Doctors less frequently restricted access to pills (48%), condoms (29%), and injectables (68%). About 50% of nurses and midwives and only 20% of TBAs restricted a client's access to condoms. Pill restrictions based on marital status were also common, at 77% of nurses, 72% of midwives, and 62% of TBAs.
Kenya (Tumlinson et al.^15^)	676 (291 public, 385 private)	58% imposed minimum age barriers for 1 or more methods. Minimum age restrictions were commonly imposed on clients seeking injectables, with large numbers refusing to offer injectables to women younger than 20 years. A significantly higher percentage of providers in private facilities imposed minimum age restrictions across all methods (e.g., 55% of private providers vs. 27% public providers for implants and IUDs).	41% restricted access to 1 or more methods based on parity. Less than 2% of providers restricted access to condoms or EC, and 60% restricted access to female sterilization based on parity. For female sterilization, 46% of providers (among those that offer sterilization and restrict on parity) required a woman to have at least 3 or more children before receiving the method.	22% of providers will not offer 1 or more methods to unmarried women. Very few providers restricted access to pills, EC, or condoms based on marital status. Approximately 10% reported that they would not provide injectables, IUDs, or implants to unmarried women, and 40% would not provide female sterilization.
Nigeria (Schwandt et al.^16^)	1,479 health facility providers, 415 pharmacists, 483 patent medicine vendors	Minimum age restrictions ranged between 70% and 93% across method and provider. Restrictions were relatively lower for condoms, EC, and pills (70%–87%), and highest for injectables and IUDs (84%–93%).	Minimum parity restrictions ranged between 3% and 65% across method and provider type. Restrictions were lowest for condoms (3%–6%), followed by EC (12%–20%). Restrictions for injectables were reported by 65% of health facility providers versus 22% of pharmacists.	Marital status restrictions ranged between 7% and 74% across method and provider type. Restrictions based on marital status were lowest for condoms (7%–10%) and EC (17%–26%), and highest for IUDs (67%) and injectables (45%–73%).
Senegal (Sidze et al.^17^)	637 (516 from public facilities, 121 from private facilities)	Minimum age restrictions were common in the public sector for the pill (57%), injectable (44%), and implant (45%). Restrictions were less common for condoms 25%) and EC (24%). Restrictions were slightly lower for private providers: pill (49%), injectable (41%), implant (38%), condom (20%), and EC (21%). On average, providers in both sectors required clients to be at least 18 for most methods.	Not reported	Between 12% and 14% of public sector providers required that a woman be married to receive the pill, injectable, or implant, and 8%–9% had that requirement for condoms and EC. In private health facilities, 21%–30% of providers did not offer unmarried women the pill, injectable, implant, or EC; 12% did not offer condoms.

Abbreviations: EC, emergency contraception; IUD, intrauterine device; TBA, traditional birth attendant.

#### Provider Bias Against Specific Populations

Bias often is directed toward specific populations or types of clients. The reproductive health community is paying increasing attention to the issue of bias toward youth. But other populations also experience notable bias, including women with HIV, women seeking abortion or postabortion care, women with disabilities, and men seeking permanent contraception. Significant literature exists regarding stigma, particularly around HIV and abortion. Such stigma contributes to biased attitudes and behavior by providers toward these populations.

Increasing attention is being paid to the issue of bias toward youth, but other populations also experience notable bias.

Some studies discuss a population group that is infrequently mentioned in the literature on provider bias—men. In noting this issue, 1 paper defined provider bias as^19^:

*the attitude of a provider who provides services only to individuals who he/she is comfortable with, or who does not feel the need to reach out to a particular group with reproductive health information with the understanding that it may not be beneficial to them.*

The authors argue that provider bias against men in sexual and reproductive health in developing countries has attracted attention only as part of wider male involvement issues.^19^ One of the major obstacles to expanding male-involvement programs is provider bias, described as programs being oriented to women^20^ and a sizable proportion of providers, whether doctors, midwives, nurses, or community workers, being women themselves and potentially uncomfortable advising and counseling men. Most of the literature around provider bias in family planning has focused on women because most services focus on women as clients, which is a function of the reality but also a possible reflection of a broader bias regarding male involvement.

### Method-Related Bias

Provider bias for or against certain methods can be related to positive or negative attitudes, inaccurate knowledge, inadequate skills, or other service-related factors, such as a method's relative ease or difficulty of administration. Attitudes seem to play out in particular as a strong bias for or against long-acting methods. Service-related factors are most often noted as a positive bias toward injectables and a negative bias toward IUDs, owing to the former being easy to administer while the latter requires a pelvic exam. Numerous studies have found bias against hormonal methods, particularly for young or nulliparous women, due to unfounded concerns about their impact on fertility. Below, we discuss some of the biases noted in the literature around specific methods. Although program-related biases may also exist—for example, if a new method is being introduced into a system, a provider might promote it more actively—we focus here on bias stemming from provider attitudes and beliefs.

Provider bias regarding methods can be related to positive or negative attitudes, inaccurate knowledge, inadequate skills, or other service-related factors.

#### LARCs: IUDs and Implants

A common perception is that provider bias is a key factor in the low use of IUDs in many countries. However, studies show a more complicated picture. A review in Ghana found demand factors and myths in the community were a greater issue and providers actually had a favorable attitude toward the method.^21^ A study in Zimbabwe looking at provider attitudes toward IUDs and HIV risk found that high proportions thought the IUD was a good method and it did not increase HIV risk for women, but they were concerned that IUD insertion put the provider at high risk of HIV infection.^22^ Providers in Kenya also had this fear of HIV acquisition, and while they were not concerned about safety or efficacy of the method for clients, they were reluctant to provide it due to it being time-consuming and challenging and their fear of potentially being blamed for any fertility problems.^23^ As access to and use of LARCs has increased, some have expressed concern of bias toward overpromotion of these methods. This concern was present, for example, in a U.S. study of users' attitudes toward or experiences with provider influence and bias regarding LARCs. These qualitative data revealed that many participants believed that providers recommend LARCs disproportionately to socially marginalized women, providing another example that shows the interaction between method and client-related bias.^24^

#### Emergency Contraception

Despite extensive evidence of its safety, emergency contraception is often perceived as unsafe or inappropriate. Some of the bias around it overlaps strongly with the bias around provision to youth, for example, believing that it leads to promiscuity. A 2015 review of improving access to emergency contraception through workforce interventions found widespread misconceptions among providers, including the belief that it was an abortifacient or that access to it would increase sexual activity among adolescents.^25^ Using survey data in Kenya and Ethiopia to explore bias around emergency contraception, Judge et al.^26^ found that counseling on and provision of emergency contraception was positively associated with providers' greater level of knowledge of the method, indicating that increasing provider knowledge can potentially contribute to offsetting some of the bias and improving access.

Some of the bias around emergency contraception overlaps strongly with the bias around provision to youth.

#### Vasectomy

Shelton and Jacobstein reported that^27^:

*providers themselves often have poor knowledge about vasectomy or bias against it, and so they fail to discuss it or provide accurate information to clients.*

Notably, the issue of bias regarding vasectomy does not arise frequently in the provider bias literature, likely in part due to the bias toward the method, its limited use in many programs, and the focus in bias literature around youth populations.

### What Are the Underlying Causes of Bias?

Many of the previously mentioned studies describe the existence of bias, but most do not go into detail about the causes. Bias can be caused by lack of accurate knowledge about the method itself or the latest normative guidance about it. Bias may also be influenced by social and cultural norms and/or affected by health systems issues including organizational culture and norms. Without a clear understanding of the causes, the risk of pursuing less effective interventions to reduce provider bias is present. For example, situational or systems factors can lead to the outcome of limiting choice, but the interventions to address these factors differ from those that could effectively address provider bias fueled by attitudes and social norms.

Bias may be influenced by social and cultural norms as well as organizational culture and norms.

The 1994–1995 study in Malawi explored providers' attitudes and beliefs in some detail, finding reservations regarding provision of family planning to youth, described earlier, as well as some general misgivings about contraceptives. Two-thirds of providers agreed with the statement “every method could be dangerous to someone” and 41% believed that contraceptive methods could have serious side effects.^12^ The authors identified 4 prime issues underlying the negative attitudes of a large number of providers, which resulted in limiting choice to clients: (1) suspecting that access to family planning is not beneficial for everyone of reproductive age; (2) harboring a deep-seated distrust of contraceptives; (3) finding the job of supplying people with contraceptives to be tiresome, unrewarding, and even disgusting; and (4) thinking that the client should not make or is not capable of making decisions about terminating childbearing on her own.

A study in Ghana used situation analysis data as a starting point to identify facilities where providers indicated high levels of imposing barriers based on parity, age, marital status, spousal consent, and other reasons.^28^ Interviewers then visited this purposive sample to probe more deeply about the reasons for these restrictions. Concerns about client safety and morals were the most often cited rationales for restricting services according to age and parity. Many providers were especially concerned that contraceptives might cause future fertility problems, and they used minimum age or parity requirements to ensure that only women of proven fertility could obtain contraceptives. Some providers believed in particular that injectable contraceptives cause permanent infertility.^28^ The authors concluded the following:

*While protecting clients' health is an admirable goal, providers who lack technical knowledge of contraception may exaggerate the dangers of various methods. In seeking to impose their personal morals on clients, providers violate basic client rights.*

Several other studies echoed the themes from Ghana and Malawi. For example, providers in Nigeria explained that one of the main reasons for an emphasis on promoting condoms among unmarried clients was due to concern about hormonal methods causing delays in pregnancy or leading to infertility. Some of this bias was also due to providers' lack of up-to-date technical knowledge, or in the case of some private-sector providers, what methods they actually provide since recommending a method they do not provide would cause a loss of business.^8^ In Lesotho, focus group discussions with clients highlighted the following frequently heard concern from providers about contraception causing infertility, as described by a married urban woman in her 30s^14^:

*At the clinic that I go to, the nurse tells young girls that she … does not want to be blamed if they became infertile …. She makes no compromise with the injectable; she bluntly refuses.*

Like all individuals in a society, providers are influenced by the social norms around them, which can lead to various biases. Sometimes, the norms are against family planning or limiting family size. A study using simulated clients in Nepal found this negative perception of limiting family size among providers, with a particular bias against poor, low-caste clients and pressure to have large families and sons^29^:

*[You] must wait for a son, even if you bear 7 or 8 daughters. You must satisfy your husband by making him the father of a son. Go on having babies until you produce a son.*

In Senegal, provider-imposed restrictions are most likely a reflection of the country's long history of restrictive family planning practices and a generally socially conservative environment.^17^

Social norms can influence a provider directly in terms of their own beliefs and also through concern about community reactions. A study of private providers in South-West Nigeria found that many providers wanted to make sure that married clients had permission from their husbands so as to avoid situations that might be harmful to their business^8^:

*[Community health workers] and providers at pharmacies and [patent and proprietary medicine vendors] were particularly likely to mention husband permission in the context of avoiding potential encounters with men upset that their wife was practicing contraception, along with a related desire to avoid creating intrafamilial conflict.*

A literature review around provider bias and adolescents described how social norms play a “formidable role” in provider bias that limits choice for adolescents. This review found that^9^:

*the most pervasive social norm was the significance of sexual abstinence before marriage. This had iterative expressions and manifestations for both clients and providers. We see this value play out in individual provider negative attitudes, and influence the degree to which clients experience discrimination based on age, marital status, and parity.*

**FIGURE 2:**
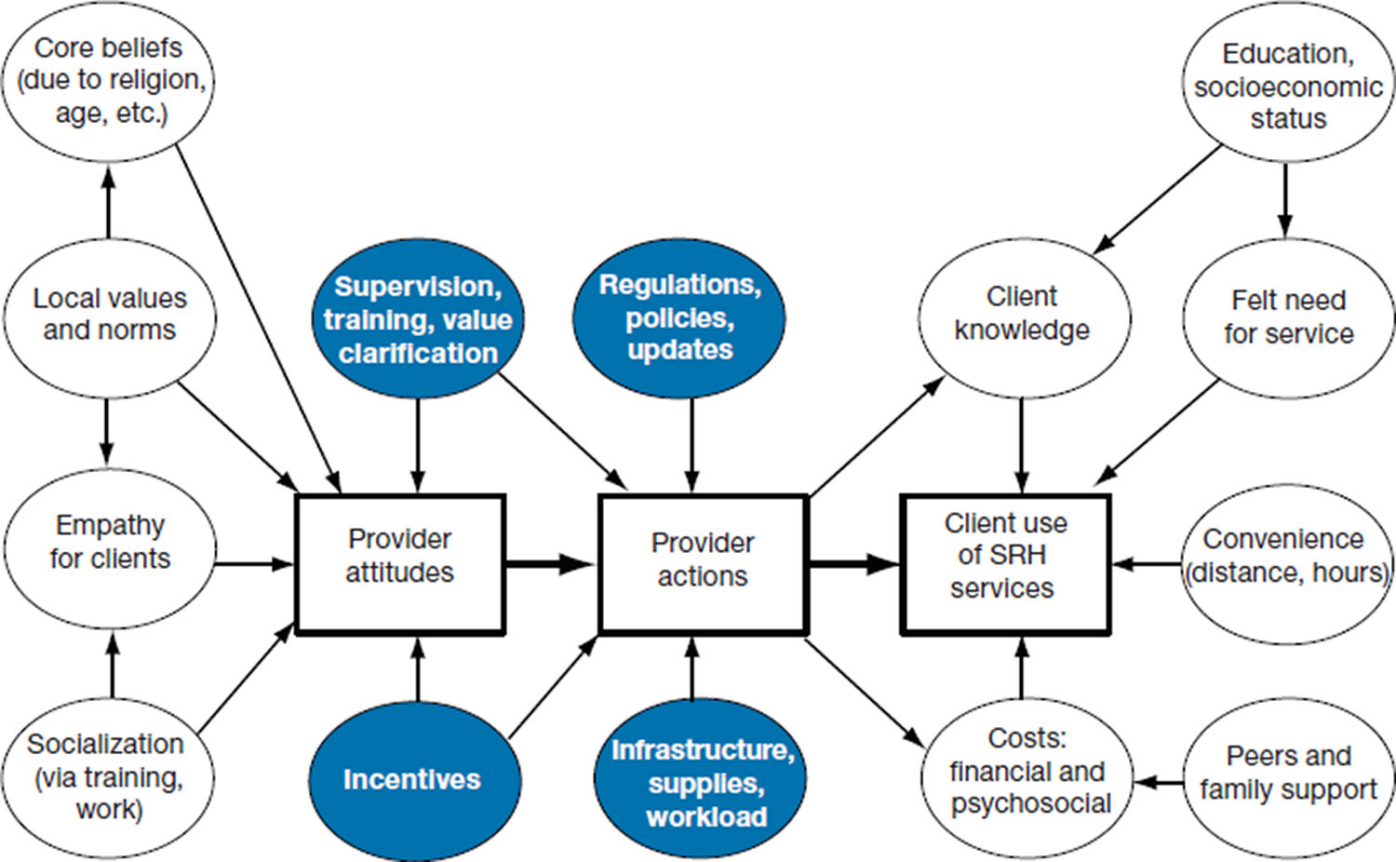
Conceptual Framework of Providers' Influence on Client Utilization of SRH Services Abbreviation: SRH, sexual and reproductive health.

Research in Senegal also noted the influence of the strong social norms against premarital sexuality for young women.^17^ Tavrow^30^ presented a useful conceptual framework of providers' influence on client utilization of sexual and reproductive health services (Figure 2), which includes the larger context of external influences, such as social norms and structural factors.

Provider bias can also be exacerbated by the hierarchical medical model. As Shelton et al. explained,^3^

*the belief that “we know better” appears in provider bias, eligibility criteria, process hurdles and regulatory constraints.*

Provider bias can also be exacerbated by the hierarchical medical model.

This attitude can limit full respect for the client and her wishes. Findings in Uttar Pradesh showed that provider-imposed restrictions stemmed from the fact that^18^:

*providers, at times, make judgments about their clients' education, [family planning] needs and ability to understand [family planning] options thereby imposing unnecessary barriers to [family planning] methods.*

A 2003 review of client-provider interactions focused on the idea of client-centered care and involving the client in decisions.^31^ The authors emphasized a client's right in making decisions, suggesting that training, along with good supervision and coaching, can make providers aware of their biases—for example, in favor of a particular method or against switching methods—that threaten clients' right to make their own decisions.

Quantitative data in Uttar Pradesh were complemented with qualitative data from in-depth interviews with 21 providers, which shed additional light on provider bias and the impact of social norms.^18^ While providers spoke about “putting choice and decision making in the hands of clients,” one of the findings from the qualitative interviews was that some providers acknowledged that they perceived many of their female clients as lacking decision making power. This perception led the providers to infer that women do not need to be offered information about their family planning options. One health worker explained^18^:

*It's like women have no say in the matter. Mostly they do what their husbands wish to do. Hence women feel that before doing anything they must take their opinion first. Whatever the men desire, happens.*

Of course, bias is not unique to health providers. A qualitative study of 50 young adult women in the United States explored users' attitudes toward and experiences with provider influence and bias around LARCs. Respondents noted the larger influences—including institutional cultures—that shape providers' contraceptive recommendations. Importantly, rather than singling out providers for being uniquely biased, several women argued that everyone in American society is affected by racial and social class biases.^24^

### What Is the Impact of Provider Bias?

A good deal of evidence indicates that providers impose restrictions that unnecessarily limit a client's choice. The discussion of impact in the literature, however, has mostly involved anecdotes and assumptions rather than extensive evidence, in part due to the difficulty in measurement. Measuring either bias or choice is a complicated endeavor, and showing a clear causal link between them is even more challenging. It is reasonable to assume that the self-reported and observed biases of providers have led to reduced access for women to a broad choice of methods. As a consequence, no methods may be available, particularly to youth, or a bias may exist toward or against certain methods. In some cases, this situation can mean use of less effective methods if a provider opts to promote only abstinence or possibly condoms to young women, which could in turn lead to higher risk of unintended pregnancy.

The 2017 literature review of provider bias in contraceptive provision to youth found that more than half of the publications they reviewed left outcomes of provider bias “up to reader interpretation or speculation.”^9^ The authors explained this lack of documentation of outcomes as being linked to methodological challenges as well as common assumptions of consequences. They also raised the issue of defining and measuring both bias and outcomes along a spectrum^9^:

*Notably, bias exists along a spectrum, from condescending or parental attitudes to inappropriate direction or denial of services to outright hostility and even violence, though outcomes of bias are seldom ranked in terms of type or severity.*

Ultimately, we want to understand how bias affects choice, but it too is challenging to measure. The fundamental importance of method choice has been validated by having “choice of methods” be 1 of the 6 elements of the seminal Bruce-Jain model of quality of care.^32^ Bertrand et al.^6^ explained that various questions have been used to capture the concept of choice in surveys, such as “Did you receive the method you wished on the day of service?”; however, no standard question is used across existing data sources. The Demographic and Health Surveys (DHS) include a question asking current users of contraception whether they were informed about other methods that could be used for contraception. Using the DHS stat compiler, results from 150 different surveys range from as low as 19.3% in Armenia in 2000 to a high of 90.5% in Burkina Faso in 2010, with an average of 67.2%. However, this measure is simply a crude indicator of choice. Truly measuring choice is complicated by the numerous structural factors that play a role, such as commodity supply or availability of trained providers.

Ultimately, we want to understand how bias affects choice, but it is challenging to measure.

At the macro level, researchers have explored method mix skew as a measure of availability of a range of methods and possibly being indicative of provider bias among other factors. An ideal method mix does not exist, but there may be reason for concern when 1 or 2 methods predominate in a given country. An analysis of method mix in 96 and 109 countries was conducted in 2006 and 2014, respectively, with the authors defining a method mix as being skewed when 50% or more of contraceptive users rely on a single method.^33^^,^^34^ Over this period, the proportion of countries with a skewed method mix decreased slightly, from 35% to 30%. The authors concluded^34^:

*Method mix skew is not a definitive indicator of lack of contraceptive choice or provider bias; it may instead reflect cultural preferences. In countries with a skewed method mix, investigation is warranted to identify the cause.*

Method mix skew can be considered a red flag warranting further exploration to see whether skew is due to lack of availability of methods, provider bias, societal preferences, or other reasons. The advantages of using skewed method mix as a red flag are that it is readily available from standardized data sets and easy to calculate.

### What Are Approaches for Addressing Provider Bias?

After noting the role of provider bias, publications often concluded that training is needed along with dissemination of updated standards and guidelines. When Shelton et al.^3^ first wrote about medical barriers in 1992, they recommended that international experts develop guidelines on family planning practices including eligibility criteria. Just a few years later in 1996, WHO published the *Medical Eligibility Criteria for Contraceptive Use* (MEC) to serve as guidance for national guidelines. A fifth updated edition was released in 2015,^35^ and an estimated 50 national programs have adopted the MEC guidance.^36^ Several studies, including those in Tanzania,^13^ Uttar Pradesh,^18^ and Ghana,^27^ concluded with the hope that revised guidelines and standards paired with training that emphasizes compliance with them would help reduce barriers. Some studies have shown an impact when guidelines are properly distributed and complemented with training and supportive supervision.^37^ However, as shown earlier, providers have regularly imposed barriers far beyond what is required in national guidelines. In addition to highlighting issues around bias, this situation raises questions about how WHO guidance and national guidelines are disseminated and how adherence to guidelines is implemented, monitored, and ensured.

Simply providing evidence about contraceptives and their safety is typically inadequate to reduce provider bias. For example, a study in Jordan found limited impact of an evidence-based medicine program on private providers' knowledge, attitudes, and practices regarding depot medroxyprogesterone acetate.^38^ The authors concluded that evidence-based medicine may not be effective as a stand-alone program targeting a family planning method with a high level of provider and consumer bias. The Kenya URHI data showed that in-service training appeared to reduce provider-imposed barriers related to parity, marital status, and third-party consent,^15^ while the data from Tanzania found that provider-determined eligibility barriers appear unrelated to whether a provider received recent in-service training.^13^ Results from the study in urban Nigeria showed the mixed and limited impact of training: while training seemed to reduce marital status bias among health facility workers, it did not help with minimum age bias or with bias among pharmacists and patent medicine vendors. The authors noted that given all the different training programs in Nigeria, knowing why this was the case was challenging, but

*It is possible that the trainings focused more on the proper techniques for administering contraceptives, the limits of what each provider is legally able to do, and the medical eligibility criteria—as opposed to socially imposed medical barriers.* ^16^

Simply providing evidence about contraceptives and their safety is typically inadequate to reduce provider bias.

A study in India looked at the impact of giving a balanced presentation of all available contraceptive methods to ensure informed contraceptive choice. With a sample of 8,077 clients, the study concluded that this approach could help to override a provider's bias by encouraging clients to make informed choice. For example, while providers saw Norplant as the first choice for 35% of the women, only 5% of women preferred and accepted Norplant, showing that providers did not always impose their bias.^39^

Carlough and Jacobstein^40^ wrote about 5 ways to address provider bias in family planning and captured some important themes: (1) provide regular evidence-based accurate information; (2) identify and use early adopters; (3) promote doing good, not just avoiding harm; (4) promote justice for all clients; and (5) support rather than blame health workers. They first describe the challenge of provider bias:

*Health workers do not walk into their client interactions as blank slates. They bring with them their personalities, cultural and socioeconomic backgrounds, understandings of “how the world works,” and biases. These biases may be against a particular method, a client characteristic or situation, or both, and they may not be immediately evident to the providers themselves.*

#### Do Not Blame Providers

Although not always the case, the literature on provider bias often contains a judgmental tone toward providers regarding their biases. Addressing bias should employ a supportive approach to change and not assign blame. This approach includes acknowledging the reality of often challenging working conditions. As Carlough and Jacobstein^40^ explained,

*Workers do not provide evidence-based, respectful family planning services in a vacuum … Health workers—especially those working in difficult conditions—need and deserve our support, particularly when asked to take on even more.*

The 1992 piece on medical barriers ended with the idea that a discussion of medical barriers is not an attack on providers, and it acknowledges that “most providers are doing what they think is best for their clients.”^3^ An important step in addressing bias is explicitly acknowledging that all people have beliefs and attitudes that can be considered biases and we must all work to ensure that these biases do not lead to behavior that has a negative impact on others.

Addressing bias should employ a supportive approach to change and not assign blame.

#### Learn From Early Adopters/Positive Deviants

Some providers can serve as mentors or role models to influence their colleagues. For example, a health facility may at first use 1 or 2 “dedicated providers” to offer clients a new method like an IUD. These providers can then mentor their colleagues to provide the same method or service.^40^ Dedicated providers for LARCs successfully expanded method choice in Zambia.^41^ Research in Cote d'Ivoire identified providers—so-called positive deviants—who had a “love for the trade,” which led to greater empathy and offering a full range of methods. While this attitude cannot necessarily be taught, such providers act as role models during training by sharing their experiences to encourage other providers.

#### Use More Comprehensive Social and Behavior Change Approaches

Over time, the response to addressing provider bias has gotten more sophisticated and comprehensive in recognizing the complicated nature of changing attitudes and behaviors that are often deeply rooted in social and cultural norms. For example, practices such as values clarification have been recognized as important parts of training. Several newer projects are tackling this issue, and lessons from this work can provide important guidance as the field aims to address this long-standing barrier more holistically.

Over time, the response to addressing provider bias has gotten more sophisticated and comprehensive.

- **The Nigeria Urban Reproductive Health Initiative 2 (NURHI 2)** is using the principle of human-centered design to address health provider bias, framing the issue as a design challenge of how to encourage providers to offer all clients the full range of methods regardless of a client's age, marital status, parity, partner consent, or socioeconomic status. Providers have received intensive training that asks them to put themselves in the shoes of clients who want to prevent pregnancy and to help them understand that regardless of their personal beliefs, their job is to help these clients obtain modern contraception. Visits to NURHI clinics have increased significantly between years 1 and 2 of the project, possibly due at least in part to this work.^42^
- ***Breakthrough ACTION and Breakthrough RESEARCH*** are 2 projects funded by the United States Agency for International Development that focus on evidence-based behavior change. Breakthrough ACTION uses a range of behavioral science approaches such as market insights, human-centered design, and behavioral economics to improve programs. For example, the project is testing interventions in Malawi to address the problem of providers not counseling clients on appropriate contraceptive options. One hypothesis is that providers rationalize incomplete counseling due to outcome bias; that is, so many women use the injectable that providers assume that must be what women want, rather than recognizing underlying structural issues that lead to that outcome. Breakthrough RESEARCH identified provider behavior change as 1 of the 2 key programmatic themes for the project's research and learning agenda. The project is developing priority research questions through a consultative process, with the following definition for provider behavior change programming^43^:

*Interventions that seek to positively influence provider behaviors to improve quality of services, improve client experiences, and increase demand for services, to increase adoption or maintenance of desired behaviors among clients and impact health outcomes.*

- ***Beyond Bias*** is funded by the Bill & Melinda Gates Foundation and is working in Burkina Faso, Pakistan, and Tanzania to address the different types of provider biases and behaviors that translate into barriers for youth access to contraceptive services. The Beyond Bias project developed a Bias Driver Tree that identified 3 categories of bias drivers, with multiple subcategories for each: biographic (attitude, abilities, experience, knowledge), situational (professional, social), and societal (beliefs/norms, law/policy).^9^

#### Be Clearer and More Proactive About Nondiscrimination

Findings in Senegal draw attention to the idea of not only having guidelines without restrictions, but also having more proactive and clear messages about the need to not restrict access based on one's own beliefs. Although current norms and protocols in Senegal do not include restrictions against youth access to family planning services, they also do not include a clear statement that young people should have unrestricted access; therefore:

*In the absence of a clear message, providers in Senegal can define their restriction criteria based on their own opinions and values regarding sexuality and contraception.* ^17^

An example of clear guidance is the *Global consensus statement for expanding contraceptive choice for adolescents and youth to include long-acting reversible contraception*, which was developed in 2016 and has been endorsed by 53 organizations.^44^ The statement cites WHO's 2015 MEC: “Age alone does not constitute a medical reason for denying any method to adolescents.”^35^ This statement has been used as a policy advocacy tool, but it could also be used directly with providers to create proactive messages through training and other means.

## CONCLUSIONS

The growing emphasis on a human rights framework in reproductive health programs makes this an opportune moment to focus on addressing provider bias to ensure the right of nondiscrimination for all clients. Ample evidence demonstrates the presence of bias, which is widely recognized as an important barrier. However, there is still a lack of an agreed-upon, clear framework for the issue that would facilitate effectively minimizing the impact of bias on access and choice. Newer approaches to address bias that have moved beyond traditional training and guidelines development to more fundamental behavior change efforts show promise, and learning from their lessons will be important. A major question will be how to scale up these approaches. Success has often come from^9^:

*a multi-faceted “kitchen sink” approach that employs as many intervention tools as available, an unscalable approach that has neither sufficiently addressed the underlying drivers of providers' biases towards youth nor led to interventions that can be systematically deployed at scale.*

The growing emphasis on a human rights framework in reproductive health programs enhances focus on addressing provider bias.

Some important steps moving forward are described below.

**Develop a Clear Definition of Provider Bias.** The field needs an agreed-upon definition, one that separates attitudes and behaviors and focuses on providing choice without discrimination. We have synthesized the common themes from the literature into a proposed working definition as a starting point:

*Provider bias refers to attitudes and subsequent behaviors by providers that unnecessarily restrict client access and choice, often related to either client and/or contraceptive method characteristics.*

It would be useful for a group like WHO to convene experts to reach consensus on a definition for the field to facilitate standard measurement and effective interventions.

**Explore Ways to Present Method Options With Minimal Bias.** Since most people—including providers—have personal biases about methods, it would be helpful to identify and promote ways to present options with minimal bias, while always ensuring that counseling begins with questions about a client's reproductive intentions and needs and ensures that a client's choice is respected. Guidance from the American Academy of Pediatrics in their 2014 policy statement on contraception for adolescents states that

*pediatricians should counsel about and ensure access to a broad range of contraceptive services for their adolescent patients. This includes educating patients about all contraceptive methods that are safe and appropriate for them and describing the most effective methods first.* ^45^

Similarly, others have suggested discussing methods in order of effectiveness, according to the WHO tiered effectiveness model, given that there are often misunderstandings by clients about a method's actual effectiveness. This approach can help ensure true informed choice and avoid the consequences of “misinformed choice.”^46^ This is only one possible approach, however, and there is currently not consensus on it.

**Monitor Bias.** Programs can explicitly monitor whether they are addressing provider bias. For example, a WHO document on monitoring human rights in contraceptive services and programs includes the following recommendation:

*Determine whether health workers have been trained in … how to ensure that users, including adolescents, can make an informed choice, including choosing to accept or not to accept a contraceptive method, without imposing their own views or using coercion (i.e. provider bias).* ^47^

**Complement Provider-Based Contraceptive Provision With Direct-To-Consumer Efforts.** While the field continues to support improvements in client-provider interactions, exploring ways to effectively get evidence-based information directly to potential clients will also be useful. This becomes particularly important with growing efforts around direct-to-consumer marketing and self-care, acknowledging that counseling is not the only way to support informed decision making.

It is critical for the family planning community to more effectively address the barrier of provider bias. Just as we ask providers not to judge a client or a contraceptive method based on their personal biases, we should not judge providers. We must work together to truly achieve the right to choice for all women and men.

## Notes

**Peer Reviewed**

**First Published Online:** September 12, 2019

**Funding:** This publication was supported by funds from USAID and WHO Department of Reproductive Health and Research, and UNDP/UNFPA/UNICEF/WHO/World Bank Special Programme of Research, Development and Research Training in Human Reproduction (HRP).
